# Dyeing of Polyester and Nylon with Semi-synthetic Azo Dye by Chemical Modification of Natural Source Areca Nut

**DOI:** 10.1007/s13659-017-0144-8

**Published:** 2017-11-28

**Authors:** Ashitosh B. Pawar, Sandeep P. More, R. V. Adivarekar

**Affiliations:** Department of Fibers and Textile Processing Technology, Institute of Chemical Technology, Mumbai, India

**Keywords:** Semi-synthetic azo dye, Chemical modification, Modified dyes, Areca nut, Ultraviolet protection factor

## Abstract

Various azo compounds (Modified dyes) have been synthesised by chemical modification of areca nut extract (epicatechin), a plant-based Polyphenolic compound to get semi-synthetic dyes. Three different primary amines namely p- nitro aniline, p-anisidine and aniline, were diazotized to form their corresponding diazonium salts which were further coupled with an areca nut extract. Preliminary characterization of the areca nut extract and the resultant azo compounds (Modified dyes) was carried out in terms of melting point, solubility tests, thin layer chromatography, UV–Visible and FTIR spectroscopy. These modified dyes were further applied on polyester and nylon fabrics and % dye exhaustion was evaluated. Dyed fabrics were further tested for their fastness properties such as wash fastness, rubbing fastness, light fastness and sublimation fastness. The results of the fastness tests indicate that, all the three modified dyes have good dyeability for polyester and nylon fabrics. The dyed fabrics were also tested for ultraviolet protection factor which showed very good ultraviolet protection.

## Introduction

Globally there is growing awareness about sustainable wet processing of textiles which requires equal consciousness towards economic, social and environmental relevance of the processes and products used therein. There are stringent environmental regulations to follow of varying degree of severity imposed by many countries in response to the hazardous effluent generated during synthesis as well as during its use in coloration due to unexhausted dyes. The toxic and allergic reactions associated with the synthetic dyes leads to the interest in the use of environment friendly dyes such as safer synthetic dyes and indisputably natural dyes. Therefore there is resurgence in the interest in the natural dyes and again textile researchers are being engrossed in experimenting with natural dyes for coloration being renewable and non-toxic [1, 2].

Having said so, natural dyes do have some inherent limitations such as poor tinctorial strength, lack of reproducibility, inferior fastness properties and un-exhausted metallic mordants in the residual dye bath posing serious challenges to the environment [3–8]. Chemical modification of the natural dyes can overcome some of these inherent challenges to make them preferred choice under prevailing scenario.

The vast quantities of plant-based Polyphenolic compounds are reported in literature as possible substitutes for synthetic phenols since they readily undergo the conventional coupling reaction with diazonium salts [9, 10]. However, relatively little research work has been published regarding the use of these plant-based Polyphenolic compounds in the synthesis of azo dyes as couplers with diazotised base [11–15].

Areca catechu contains diverse phenolic compounds, including condensed tannins, hydrolysed tannins, lignans, stilbenes, flavane, flavonoids (mainly catechin and epicatechin), anthocyanin, and simple phenolic acids. Areca seed extracts are useful nutritional antioxidants for the nutraceutical industry with remarkable benefits to human and animal health. Epicatechin and syringic acid are the two major phenolic compounds present in the areca nut extract [16–18], which are structurally represented as, 

Structures of (a) syringic acid and (b) epicatechin

In the present study, an attempt is made to chemically combine a plant-based Polyphenolic compound from Areca Catechu, by coupling it with diazonium salts of three different primary amines, to modify its substantivity and tinctorial capacity to overcome inherent limitations of Areca catechu as a natural dye.

## Results and Discussion

The purity of all three dyes was analysed by TLC method using Chloroform: Methanol (1:4) as the solvent system. The dyes produced a single colour spot on silica chromatography plates, their respective R_f_ value were shown in Table 1. Furthermore the solubility of modified dyes in different solvents were given in Table 2.

**Table 1:** Physical properties of areca nut extract and modified dyes

Legend	Diazotising component	% Yield	R_f_ value	Melting point (°C)
Source (areca nut extract)	–	40	–	115
AN-1	p-nitro aniline	88	1.9	228
AN-2	p-anisidine	80	2.1	220
AN-3	aniline	74	1.3	245

**Table 2:** Solubility data of modified dyes

Legend	Water	Methanol	Ethanol	Acetone	Ethyl acetate	Chloroform	DMF	DMSO
Source	I	I	I	I	I	I	VS	VS
AN-1	VSS	SS	SS	S	SS	VSS	VS	VS
AN-2	VSS	SS	SS	S	SS	VSS	VS	VS
AN-3	VSS	SS	SS	S	SS	VSS	VS	VS

*I* insoluble, *VSS* very slightly soluble, *SS* sparingly soluble, *S* soluble, *VS* very soluble

### FTIR Analysis

**Fig. 1:**
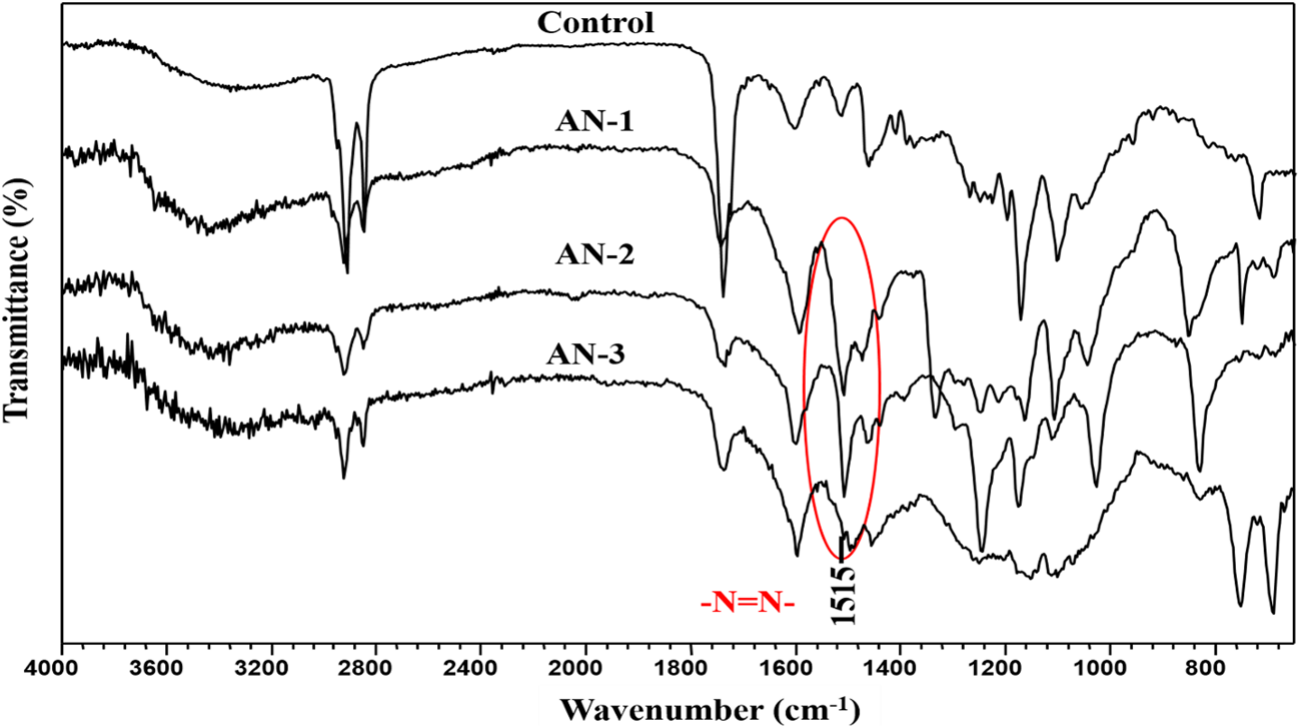
FT-IR spectra for extracted areca nut (control sample) and modified dyes

FTIR analysis was carried out to assess the areca nut extract sample and respective chemical modification occurred in modified dyed sample. The FTIR spectra obtained for extracted areca nut (control sample) and AN-1, AN-2, AN-3 are shown in Fig. 1.

In Fig. 1, the FT-IR spectra for areca nut (control sample) shows the presence of benzene ring in aromatic compounds as indicated by the sharp peak with medium intensity in the range of 1615–1590 cm^−1^. The presence of C–O–C stretch in aromatic compounds is indicated with strong intensity peak in the range of 1280–1180 cm^−1^. The presence of CH–O–H in cyclic alcohol is indicated with sharp band at 1058.85 cm^−1^. Also the presence of C–OH in alcohols is indicated by (C–O) stretch band at 1178.43 cm^−1^. Thus the FT-IR spectrum for control sample shows that the compound is Polyphenolic substance with characteristic bands which are amenable for chemical modification. Whereas the FT-IR spectra for all the three modified dyes AN-1, AN-2, AN-3 respectively shows the presence of azo groups in the range of 1450–1520 cm^−1^, which is absent in the FTIR of control sample indicating the successful chemical modification of areca nut extract.

### Spectral Properties of Dyes

These dyes were subjected to spectrophotometric analysis for assessment of the λ_max_. The results of the spectrophotometric measurements are listed in Table 3. The absorption maxima of the synthesised dyes which produces Lemon yellow, Honey gold and Golden yellow shades on fabrics was found to be 431.0, 385.5 and 385.0 respectively (Table 4).

**Table 3:** Spectral properties of dyes

Dye	λ_max_	Shades on fabrics
AN-1	431.0	Lemon yellow
AN-2	385.5	Honey gold
AN-3	385.0	Golden yellow

**Table 4:** Colour appearance of modified dyes and its respective dyed samples

Dye	Dye in powder form	Colour of the dye in powder form	Dyed polyester	Dyed nylon
AN-1		Reddish brown		
AN-2		Orange		
AN-3		Black brown		

The colour data shown in Table 5 is in agreement with the spectral data of all the three modified dyes. The L* values were higher in case of dyed polyester corresponding to lighter shades whereas the L* values were found to be lower in case of dyed nylon corresponding to darker shades. The values were in a positive coordinates in terms of a* and b* which corresponds to redness and yellowness of dyed fabrics in terms of tonal variation. Also the % exhaustion of all the dyes on the fabrics is appreciable. The higher exhaustion on the nylon fabric was expected due to its relatively open structure being knitted fabric as compared to polyester which is woven fabric, making it easily accessible for dyeing. Consequently, the diffusion of the dye within the fabric proceeds rapidly under the given dyeing condition. Hence, the rate of diffusion of the dye molecules into the fabric is higher, which increases the exhaustion value [19–21].

**Table 5:** L*, a*, b* values of dyes (1% shade) on dyed polyester and nylon fabric

Dye	Fabric type	L*	a*	b*	Exhaustion (%)
AN-1	Polyester	67.84	4.596	21.509	72
	Nylon	43.563	16.337	22.923	82
AN-2	Polyester	59.755	10.664	17.819	84
	Nylon	38.959	15.787	17.215	87
AN-3	Polyester	67.289	5.049	20.39	82
	Nylon	45.826	10.089	21.335	89

### Colour Build-up of Dye

**Fig. 2:**
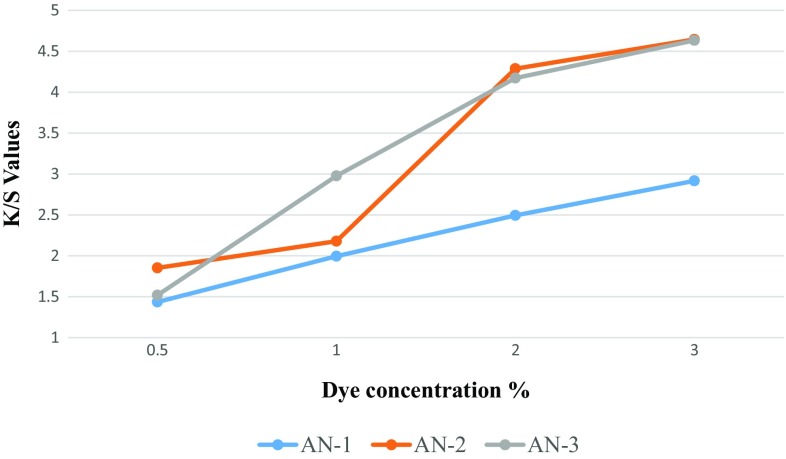
Concentration of dye solution versus K/S values data for dyed polyester fabric

**Fig. 3:**
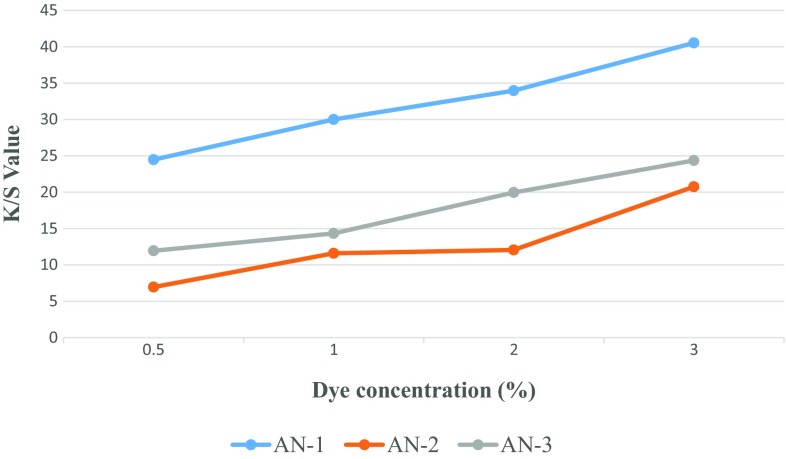
Concentration of dye solution versus k/s values data for dyed Nylon fabric

Figures 2 and 3 show the dye uptake of the dyes in terms of K/S values at various concentrations of dye such as 0.5, 1, 2 and 3%. All the three dyes exhibited good shade build-up on the polyester and nylon fabrics. However, it has been observed that K/S values of dyed nylon fabrics are higher than that of the dyed polyester fabrics, which is attributed to the higher % exhaustion of dyes on the nylon fabric as compared to the % exhaustion of dyes on polyester fabric.

## Fastness Properties of Dyed Fabrics

Results for wash, rubbing and light fastness are summarised in Table 6 and the results for sublimation fastness are summarised in Table 7. The results clearly indicate excellent all round fastness properties for all the three modified dyes. The results of the fastness tests of the dyed fabrics show that the dyes have good dyeability for polyester and nylon fabrics.

**Table 6:** Results for wash, rubbing and light fastness

Dye	Fabric type	Wash fastness		Rubbing fastness		Light fastness
		A	B	Dry rubbing	Wet rubbing	A
AN-1	Polyester	3	4	5	4–5	5–6
	Nylon	4	4	4–5	4	5
AN-2	Polyester	3–4	3–4	4–5	4–5	6
	Nylon	4	4	4–5	4	5
AN-3	Polyester	4	4	4–5	4	5–6
	Nylon	3–4	3–4	4	3–4	5

*A* change in colour, *B* staining on adjacent fabric

**Table 7:** Results for sublimation fastness

Dye	Fabric type	Sublimation fastness					
		150 °C		180 °C		210 °C	
		A	B	A	B	A	B
AN-1	Polyester	4–5	5	4–5	4–5	4	4
	Nylon	5	5	4	4	3	3
AN-2	Polyester	4	4	4–5	4–5	3	3
	Nylon	4–5	4–5	4	4	3–4	3–4
AN-3	Polyester	5	5	5	5	4	4
	Nylon	4–5	4–5	4	4	3	3

*A* change in colour, *B* staining on adjacent fabric

### Ultraviolet Protection Factor (UPF)

Textile Clothing can protect the skin from harmful UV radiations because the fabric can reflect, absorb and scatter these rays and reduce the amount of radiation transmitted through it. UV protection provided by a textile material is measured in terms of its ultraviolet protection factor (UPF). It is defined as a ratio of average effective UV irradiance calculated for unprotected skin to the average UV irradiance for skin protected by the test fabric. Below is the (Table 8) classification scheme for UPF rating published in 1996; the Australian and New Zealand standard for evaluation and classification of sun protective clothing (AS/NZ 4399), considered as the benchmark for industry [22–25].

**Table 8:** Classification scheme for UPF rating

UPF rating	% UVR transmitted	Protection category
15–24	6.7–4.2	Good
25–39	4.1–2.6	Very good
40–50+	≤ 2.5	Excellent

**Fig. 4:**
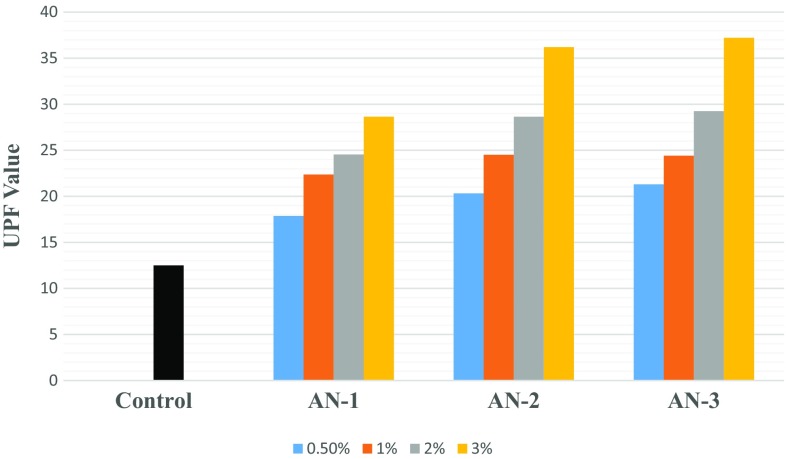
Results for ultraviolet protection factor (UPF Value) for dyed polyester fabric

**Fig. 5:**
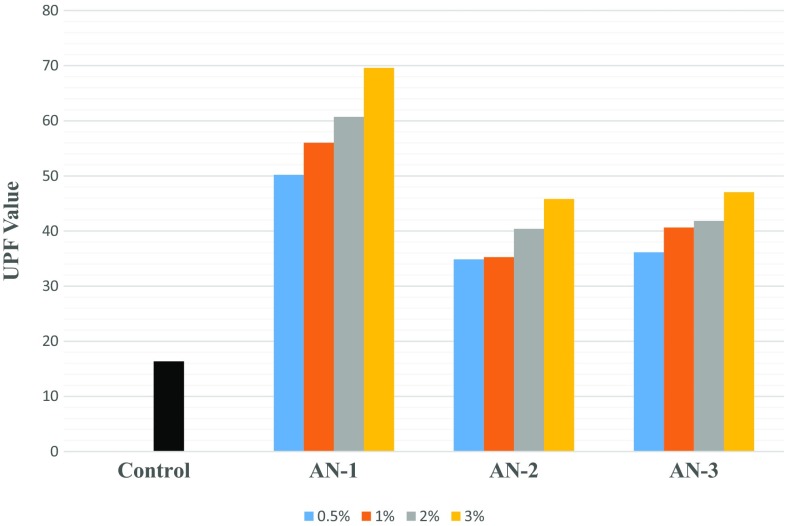
Results for ultraviolet protection factor (UPF) for dyed Nylon fabric

Figures 4 and 5 show the results for ultraviolet protection factor of control (undyed polyester and nylon fabrics), dyed polyester and dyed nylon fabrics. With the increase in the dye concentration for all the three modified dyes, dyed polyester exhibits very good UPF rating as UPF value is in the range of 20–39 which indicates the transmittance of 4.1–2.6% (i.e. blocking 95.9–97.4%) of the UV radiation. Similarly, dyed nylon exhibits excellent UPF rating as UPF value is in the range of 40–50+ which indicates the transmittance of ≤ 2.5% (i.e. blocking 97.5%) of the UV radiation. So, all the three modified dyes impart excellent ultraviolet Protection to the textile material. UPF values are found to be higher in case of dyed nylon fabric than that of dyed polyester. This may be attributed to the fact of higher % exhaustion of dye and compact structure of nylon fabric as compared to polyester.

## Experimental Section

### Materials and Methods

#### Materials

*Source* Areca Nut Extract.

*Substrate used* Industrially ready for dyeing (RFD) 100% woven polyester and knitted nylon fabrics were purchased from local source in Mumbai.

*Chemicals used* p- nitro aniline, p-anisidine, aniline, sulphuric acid, hydrochloric acid, sodium nitrite, N, N-dimethyl formamide and acetone, all LR grade, supplied by SD fine chemical ltd. (Mumbai, India).

## Experimental

Areca nut was grounded into powder form. 10 g of this areca nut powder was extracted with 350 ml of ethanol for 6 h, with the help of soxhlet apparatus. The extract was dried by evaporating the solvent on vacuum rotary evaporator to get powder of extract. The yield obtained was 40%. The melting point of the powder extract was measured and TLC was carried out to determine its purity. This extract was used as a coupler.

### Diazotisation

In this work three different primary amines are used namely p- nitro aniline, p-anisidine and aniline. These amines were firstly diazotized to form there diazonium salts by using reported methods [26].

### Coupling

The 0.5 g of extracted areca nut powder was dissolved in 2 N Sodium hydroxide solution with constant stirring. This solution was used as a coupler. The respective cold diazonium salt solution was added drop wise to the mixture of coupling component with continued stirring for 2–3 h in ice bath. The resultant dyes were then filtrated on Buchner funnel using suction filtration, washed with cold water and dried at 60 °C in an oven. These dyes were further analysed for purity using TLC technique and percentage yields were calculated.

## Application of Modified Dyes

These modified dyes were used for dyeing of 100% polyester and nylon fabrics. 1% stock solution of modified dye was prepared by dissolving it in a DMF solvent. Dyeing of polyester was carried out by High Temperature High Pressure Method (HTHP) at 130 °C for 45 min by keeping material to liquor ratio 1:30 and maintaining pH of 4.5–5.5 using Glacial Acetic acid. The dyed fabrics were subjected to reduction clearing treatment at 70 °C for 20 min with 2 gpl NaOH and 2 gpl sodium hydrosulphite together keeping material to liquor ratio of 1:30. The dyed samples were further washed and soaped using 1 gpl non-ionic surfactant solution.

Similarly dyeing of nylon fabric was carried out at boil for 60 min, keeping material to liquor ratio 1:30 at pH of 4.5–5.5, followed by subsequent washing and soaping. Dyeing’s obtained were then evaluated for colour measurement and fastness properties.

### Colour Measurements

Dyed fabrics were simultaneously evaluated in terms of CIELAB colour space (L*, a* and b*) values and colour strength in terms of K/S by reflectance method using 10^0^ standard observer. The absorbance of the dyed samples was measured on Rayscan-Spectrascan 5100+ equipped with reflectance accessories.

In general, the higher the K/S value, the higher the depth of the colour on the fabric. L* corresponding to the brightness (100 represent white, 0 represents black), a* corresponding to the red-green coordinate (+ ve represents red, − ve represents green) and b* corresponding to the yellow–blue coordinate (+ ve represents yellow, − ve represents blue). As a whole, a combination of these values enables one to understand the tonal variations.

The K/S values were determined using expression;

1 $$\frac{\text{K}}{\text{S}}= \frac{\left({1-\text{R}}\right)^{2}}{{\text{2R}}}$$

where, R is the reflectance at complete opacity; K is the Absorption coefficient and S is the Scattering coefficient.

### Determination of Percent Dye Exhaustion

Exhaustion is the total amount of dye taken up by dyed fabric. It was determined by measuring the optical absorbance of the dye bath before and after dyeing process. The absorbance of diluted dye solution was measured at the wavelength of maximum absorption using UV/Vis spectrophotometer.

The percent dye exhaustion (% E) was then calculated using the following equation;

2 $${\text{\% E}} = 100 \times \left( {1 - \frac{{{\text{A}}1}}{\text{Ao}}} \right)$$

where, Ao and A1 are the absorbances of the dye solutions before and after the dyeing process respectively.

### Fourier-Transform Infrared Spectroscopy

Fourier-transform infrared spectroscopy (SHIMADZU FTIR-8400S) was used to confirm the chemical modification of areca nut extract measuring reflectance spectrum of a sample. In this analysis extracted areca nut (control sample) and dye samples formed by coupling it with diazotised amines were analysed to confirm the chemical modification of areca nut extract as natural dye.

### Colour Fastness Testing

Fastness properties of the dyed fabrics were evaluated. Wash fastness testing was done by the standard method ISO105-C03.Rubbing fastness of the dyed samples was evaluated on automatic Crockmeter using the standard ISO105-X12 method. The light fastness of the dyed samples was tested on Q-Sun Xenon Test Chamber using the AATCC 16-2004 method. Sublimation fastness of the polyester dyed samples was tested on Sublimation tester using the ISO 105-F04 method. The shade change, together with staining on adjacent fabrics, was rated according to appropriate SDC grey scales.

## Conclusion

Chemical modification of natural dye; Areca Nut was done successfully using the diazo-coupling reaction of areca nut extract to get semi-synthetic dyes with appreciable yield for in tune for commercial exploitation. The physical properties of the azo compounds (modified dyes) such as melting points, R_f_ values, UV–Visible and FTIR spectroscopic results show that, obtained modified dyes are fairly pure compounds acceptable for dyeing of textiles. All the three modified dyes were successfully applied on polyester and nylon fabrics with appreciable depth and shades with excellent overall fastness properties. The dyeing in addition to performance properties also found to give very good ultra violet protection factor. Thus, it can be said that chemical modification of natural dye can be used to overcome the inherent limitations of natural dyes such as poor tinctorial strength, lack of reproducibility due to varying purity and inferior fastness properties for the bulk scale application in absence of mordants.
